# Duodenum edema due to reduced lymphatic drainage leads to increased inflammation in a porcine endotoxemic model

**DOI:** 10.1186/s40635-022-00444-9

**Published:** 2022-05-02

**Authors:** Silvia Marchesi, Anders Larsson, Göran Hedenstierna, Mohammed Abujazar, Håkan Ahlström, Miklós Lipcsey

**Affiliations:** 1 Hedenstierna Laboratory, Department of Surgical Sciences, Uppsala University, Uppsala, Sweden; 2 Anaesthesiology and Intensive Care, Department of Surgical Sciences, Uppsala University, Uppsala, Sweden; 3 Hedenstierna Laboratory, Department of Medical Sciences, Uppsala University, Uppsala, Sweden; 4 Centre for Medical Imaging, Uppsala University Hospital, Uppsala, Sweden; 5 Department of Radiology, Uppsala University, Uppsala, Sweden

**Keywords:** Abdominal edema, Lymphatic drainage, Abdominal inflammation, Abdominal perfusion, Magnetic resonance, DW-MRI, Intra-abdominal pressure

## Abstract

**Background:**

Interventions, such as mechanical ventilation with high positive end-expiratory pressure (PEEP), increase inflammation in abdominal organs. This effect could be due to reduced venous return and impaired splanchnic perfusion, or intestinal edema by reduced lymphatic drainage. However, it is not clear whether abdominal edema per se leads to increased intestinal inflammation when perfusion is normal. The aim of the presented study was to investigate if an impaired thoracic duct function can induce edema of the abdominal organs and if it is associated to increase inflammation when perfusion is maintained normal. In a porcine model, endotoxin was used to induce systemic inflammation. In the Edema group (*n* = 6) the abdominal portion of the thoracic duct was ligated, while in the Control group (7 animals) it was maintained intact. Half of the animals underwent a diffusion weighted-magnetic resonance imaging (DW-MRI) at the end of the 6-h observation period to determine the abdominal organ perfusion. Edema in abdominal organs was assessed using wet–dry weight and with MRI. Inflammation was assessed by measuring cytokine concentrations in abdominal organs and blood as well as histopathological analysis of the abdominal organs.

**Results:**

Organ perfusion was similar in both groups, but the Edema group had more intestinal (duodenum) edema, ascites, higher intra-abdominal pressure (IAP) at the end of observation time, and higher cytokine concentration in the small intestine. Systemic cytokines (from blood samples) correlated with IAP.

**Conclusions:**

In this experimental endotoxemic porcine model, the thoracic duct’s ligation enhanced edema formation in the duodenum, and it was associated with increased inflammation.

## Introduction

Edema—the increase of extravascular fluid in tissues—can have multiple causes, the most frequent being glycocalyx degradation induced by ischemia–reperfusion syndrome or sepsis [1], reduced oncotic pressure [2] and/or enhanced hydrostatic pressure in the venous or lymphatic compartment [3]. The lymphatic compartment is responsible for the drainage of excessive extracellular fluid and it increases its activity when other causes are present [4].

Most of the lymph drained from the abdomen is collected in the cisterna chyli [5] and then drained to the thoracic duct, which generates from the cisterna right below the diaphragm, it passes through it and, following the course of aorta on its left side, reaches and flows into the left subclavian vein. A smaller amount of the lymph drained from the lower part of the body is collected by a number of small lymphatic vessel that pass through the diaphragm and reconnect with the thoracic duct at a more cranial level.

The lymphatic drainage from the abdominal compartment can be impaired by multiple factors, such as malignancy affecting lymph nodes, massive surgery or trauma. Previous studies showed that high pressure in the thoracic compartment produced during mechanical ventilation [6, 7] with high PEEP (positive end-expiratory pressure) level [8, 9] or high inspiratory pressure and tidal volumes reduces the thoracic duct flow and increased edema and inflammation of abdominal organs. If the increased inflammation was caused by the increased edema or by other factors, as the reduced perfusion related to the impaired venous return, was not clarified. To the authors’ knowledge, there are no studies that investigated the effect of an acute abdominal lymphatic drainage impairment and the possible consequent increase of edema in the abdominal organs.

The presented experimental study was set out to test the hypothesis that the ligation of the thoracic duct increases edema in the abdominal organs and that edema augments inflammation. The hypothesis was tested comparing abdominal inflammation and perfusion in two groups of animals, in one of whom the thoracic duct was ligated. The hemodynamic was maintained as nearest as possible to normality in both groups by vasoactive infusion. Abdominal perfusion was assessed by diffusion weighted-magnetic resonance imaging (DW-MRI) as described by us previously. [10].

## Methods

The Animal Research Ethical Committee of Uppsala University approved the study (nr C 145/14). The study was performed according to Arrive 2.0 Guidelines [11].

Thirteen piglets, between two and three months old and with a weight between 22.5 and 25.7 kg, were studied.

All animals were given an endotoxin infusion to induce a septic-like state. They were randomized into two groups. In one group—the Edema group—the abdominal tract of the thoracic duct was ligated a couple of centimeters from its passage into the diaphragm, while the other group—the Control group—received a similar surgery to identify the thoracic duct, which was maintained intact.

A 1:1 randomization was performed using the random numbers generator application on GraphPad software (version 8.4.2.)

### Preparation

Animals were pre-medicated with Zoletil Forte™ (tiletamine and zolazepam) 6 mg kg^−1^ and Rompun Vet™ (xylazine) 2.2 mg kg^−1^ i.m. After around 5 min, the animals were placed supine on an operating table, a bolus of fentanyl 10–20 µg kg^−1^ was given through a cannulated ear vein and a tracheotomy was performed inserting a 7 mm ID endotracheal tube (Mallinckrodt Medical, Ireland).

Ventilation was started in volume-control mode by a Servo-I ventilator (Maquet, Sweden) with a tidal volume (*V*_T_) of 6 mL kg^−1^, inspiratory:expiratory ratio (*I*:*E*) 1:2, FiO_2_ 0.5, respiratory rate (RR) 25 cycles/min and PEEP 5 cmH_2_O.

Anesthesia was then maintained with a continuous iv infusion of ketamine 30 mg kg^−1^ h^−1^, midazolam 0.1 mg kg^−1^ h^−1^ and fentanyl 3.75 µg kg^−1^ h^−1^. After checking that anesthesia was sufficient to prevent responses to painful stimulation, muscle relaxation was added as a continuous i.v. infusion of rocuronium 3 mg kg^−1^ h^−1^. Thirty ml kg^−1^ Ringer’s Acetate were administered during the preparation to both groups.

In all animals, a triple-lumen thermistor-tipped balloon catheter (Swan–Ganz catheter, 7 Fr) was placed in the pulmonary artery from the right external jugular vein. Through the same access, a central venous catheter was inserted. A neck artery was cannulated and a second triple-lumen thermistor-tipped balloon catheter was placed in the hepatic vein from the left internal jugular vein (positioning was ascertained by fluoroscopy). A 20 cm laparotomy was performed at 3–4 cm left from the median abdominal line, then spleen and intestine were displaced to reach the aorta, on the left side of which the thoracic duct is normally positioned. In the Edema group, the duct was identified and ligated with a silk suture thread. The intestine was put back in place and the spleen was exposed for the cannulation of the splenic vein through a Seldinger technique, a 4 Fr catheter was positioned into the portal vein via the splenic vein. The spleen was carefully repositioned in the abdomen and peritoneum, muscle layers, and skin were sutured.

**Fig. 1:**
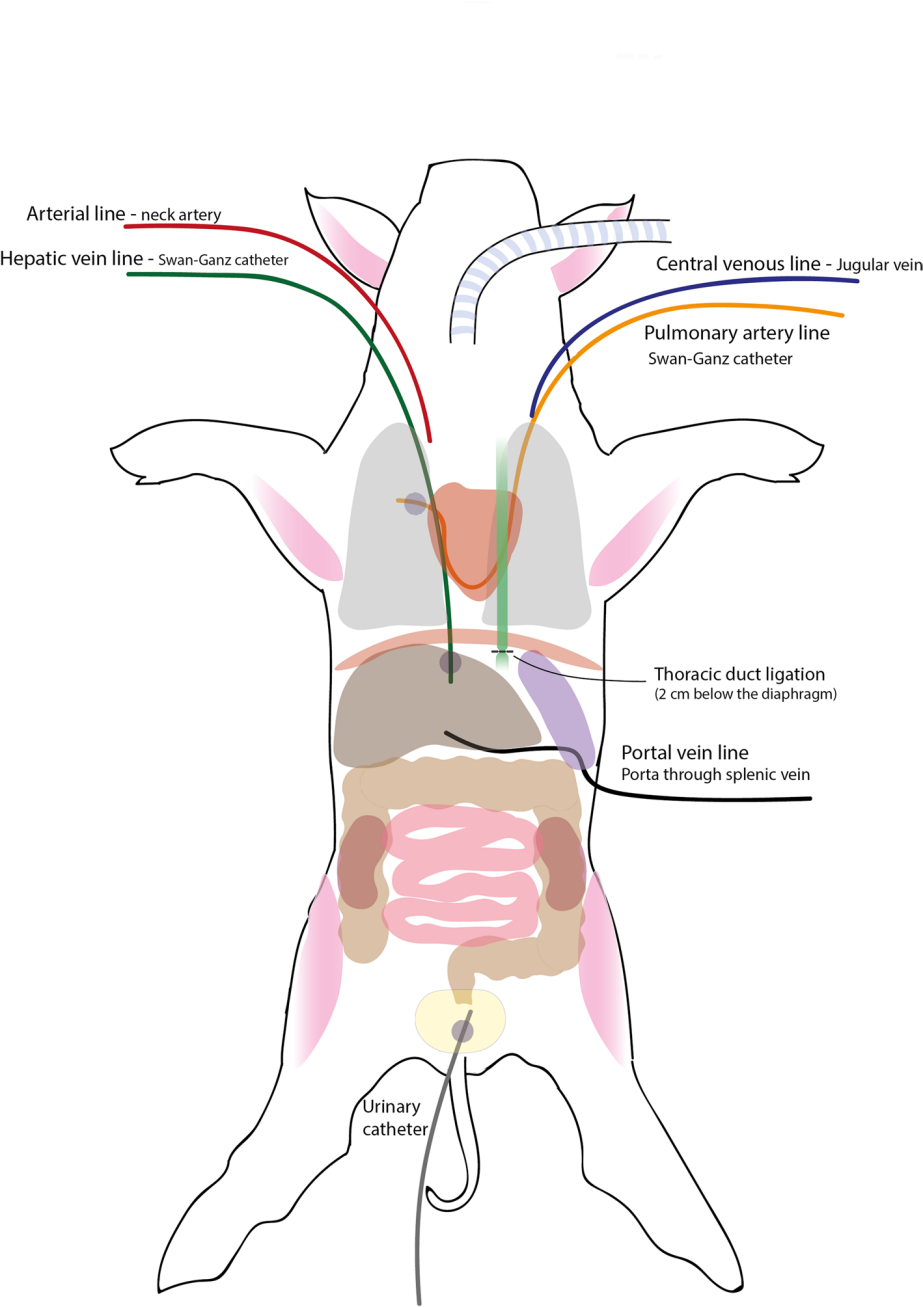
Preparation setting. Five vascular catheters are represented in the image: an arterial line, a central venous line, a hepatic vein line, a portal vein line and a pulmonary artery line. From four of the lines (excluding central venous line) blood samples were taken at baseline and hourly during the observation for blood gas analysis and cytokines concentration assessment. In the image the thoracic duct is represented in green and the level of ligation of the Edema group is reported. The abdominal organs analyzed in the study are all represented in the figure (lungs, small intestine, colon, liver, spleen and kidneys)

In the Control group we performed similar surgery, but once the thoracic duct was identified, we proceed directly with the cannulation of the portal vein. A schematic drawing of the animals’ preparation setting is shown in Fig. 1.

The catheters were used for blood sampling and pressure measurements when appropriate.

Cardiac output was measured by thermo-dilution.

A bladder catheter was inserted to collect urine and to measure intra-abdominal pressure (IAP). Abdominal perfusion pressure (APP) was calculated as:

MAP (mean arterial pressure) – IAP (intra-abdominal pressure) [12, 13].

### Protocol

After baseline measurements, an endotoxin infusion was started (Sigma, Lipopolysaccharide from *Escherichia Coli*, 0111:B4, L2630-100 mg, 129K4025). The infusion rate was 15 μg kg^−1^ h^−1^ for 2 h, then decreased to 5 μg kg^−1^ h^−1^.

A noradrenaline infusion was titrated to maintain MAP equal to or above 65 mmHg as recommended by the guidelines for resuscitation in the Third International Consensus Definition for Sepsis and Septic Shock [14].

As soon as a stable condition was reached (most of the time during the first hour of observation), PEEP was gradually increased to 15 cmH_2_O in both groups.

A higher amount of fluids (around 500 ml in total) was administered to the Edema group, in order to magnify the effect of the thoracic duct ligation.

After 6 h of endotoxin infusion, four animals per groups were taken to the magnetic resonance imaging (MRI) Research unit for image acquisition, while the rest of the animals were euthanized by the injection of a KCl bolus. The animals examined with MRI, were killed once back in the laboratory, using the same method.

General anesthesia was maintained during the imaging acquisition, while endotoxin was discontinued.

During autopsy, samples from the lungs (upper and lower lobes of both), the duodenum, the colon, the liver, the spleen and both kidneys were taken and used to measure wet–dry weight, immunohistological analysis (cytokines concentration measurement), and histopathological assessment.

### DW-MRI

Diffusion-weighted MRI was performed on a Philips 1.5 T scanner. Settings were: FOV 300 mm, 34 slices, thickness 8 mm, repetition (TR) and echo time (TE) of 1500 ms and 82 ms. Respiratory triggering was used as well as three diffusion sensitizing directions, i.e., 9 *b*-values 0, 20, 50, 75, 100, 200, 300, 600, 900 s/mm^2^.

The apparent diffusion coefficient (ADC) was measured; it is a measure of the magnitude of diffusion (random movement of water molecules), and was consider as a proxy for edema, (unit µm^2^/ms).

It is obtained by applying a mono-exponential model [15].

Using the intravoxel incoherent motion method (bi-exponential model), the *f *value, defined as the contribution of perfusion to the global movement of water molecules in a voxel [16], was calculated.

Regions of interest (ROIs) were drawn for edema and perfusion measurements in four target organs: liver, kidneys, intestine and spleen. For each organ four to six ROIs were drawn and the mean value of the ADC and *f *value was calculated. A goodness of fit was calculated to assess the quality of the measurements and ROIs with a goodness of fit lower than 95% were discarded.

### Data collected

#### General data

Hemodynamic and respiratory data were collected at the baseline and every hour during the entire observation time (6 h) (see Tables 1 and 2 for a comprehensive list).

Arterial pressure, pulmonary arterial pressure, central venous pressure and oxygen saturation were monitored continuously.

Intra-abdominal pressure was measured following the instruction of the World Society of the Abdominal Compartment Syndrome [17], through the bladder catheter and injecting a volume of 10 ml in the bladder (instead of 25 ml volume, recommended for human adults) at baseline and at every hour.

At baseline and every hour during the observation time, blood gas analyses were performed on samples taken from 4 different vascular beds: artery, pulmonary artery, portal vein and hepatic vein.

### Edema

Edema was assessed by comparing wet and dry weight in the lungs (samples were taken from the upper and lower lobes in both lungs), the duodenum, the colon, the liver, the spleen, and both kidneys.

ADC was also used as an edema assessment of the small intestine, the liver, the spleen, and the kidneys. Hemoglobin concentration was used as a surrogate of the hemoconcentration produced by the extravasation of plasma.

The abdominal circumference of the animals was measured at the baseline and after 6 h. The difference between the circumference measured at the end of the experiment and the baseline was used as an approximative assessment of edema and ascites amount in the abdomen.

An edema score (from 0 to 4) was given to lung (lower and upper lobes), small intestine and colon tissue samples by the veterinary pathologist on histopathological samples.

### Perfusion

Perfusion was assessed using DW-MRI data [16]. *f *value was compared between the groups as a measurement of perfusion in the small intestine, the liver, the spleen, and the kidneys.

### Inflammation

Samples from the duodenum, colon, kidneys, liver, and spleen were used to perform histopathological analyses of inflammation. The main features analyzed to produce the histopathological score in intestinal samples were: number and type of leukocytes, site of leukocytes localization (surface of the villi, side of the villi, or the crypts), type, intensity and extension of damage (necrosis, exfoliation, degeneration, apoptosis or erosion) [18]. In parenchymatous organs the score included the evaluation of leukocytes’ infiltrates, the presence of hemorrhage and general damages to the organ structure. A veterinary pathologist, blinded to the protocol, analyzed the samples.

Inflammation was also assessed by measurement of interleukin-6 (IL6), tumor necrosis factor α (TNFα) and interleukin 1 (IL1) concentration (by ELISA test) in blood from different vascular beds (artery, pulmonary artery, portal vein, and hepatic vein) at baseline and at the end of the observation time, and ascites and organs samples (the lungs—upper and lower lobes, the duodenum, the colon, the liver, the spleen and both kidneys).

The tissue samples were taken, as close as possible, in the same anatomical region as had previously been studied by DW-MRI.

Since the observation time in the study was short, 6 h, IL-6, TNFα and IL1 were selected for analysis, being among the earliest pro-inflammatory cytokines produced in sepsis [19]. A biologist, blinded to the protocol, performed the biochemical tests.

### Statistical analysis

Data are presented as median and range. Based on distribution (Shapiro–Wilk test), comparisons between the two groups were performed using Student’s *t*-test or Mann–Whitney test and, when an analysis of multiple time points was performed, using ANOVA and multiple *t*-tests (correction for multiple comparison using Bonferroni–Dunn method). Pearson’s or Spearman’s coefficient were calculated to correlate different variables.

Cytokines and lactate concentrations as log-normally distributed data were log-transformed before analysis.

*p* < 0.05 will be defined as statistically significant.

Statistical analysis was performed using Prism version 8.4.2 (GraphPad software); and R (open source software, version 3.6.3).

## Results

A total of 13 animals were included in the analysis, randomized into the two previously described groups. Six animals were randomized into the Edema group and seven into the Control group.

Only 3 animals out of 13 were male, 2 of them were randomized in the Control group and one in the Edema group.

The average weight of the animals was 23.5 kg.

### General data

Hemodynamic, respiratory and blood gas data are presented in Tables 1 and 2.

**Table 1:** Hemodynamics

	Baseline	1st hour	2nd hour	3rd hour	4th hour	5th hour	6th hour	ANOVA (*p* value)
*Edema group*								
MAP (mmHg)	82 ± 5	85 ± 10	68 ± 11	71 ± 10	82 ± 12	86 ± 13	81 ± 14	*0.76*
SAP (mmHg)	97 ± 10	103 ± 11	90 ± 8	92 ± 7	101 ± 8	107 ± 10	103 ± 10	*0.35*
DAP (mmHg)	62 ± 7	69 ± 8	52 ± 10	57 ± 12	67 ± 14	72 ± 15	66 ± 16	*0.97*
PAPmean (mmHg)	19 ± 3	30 ± 6	31 ± 2	39 ± 5	40 ± 3	38 ± 3	37 ± 5	*0.59*
PAOP (mmHg)	11 ± 2	13 ± 2	16 ± 4	14 ± 3	14 ± 1	15 ± 1	16 ± 1	*0.78*
CVP (mmHg)	8 ± 2	9 ± 2	11 ± 2	11 ± 1	11 ± 1	12 ± 1*	14 ± 3*	*0.08*
IAP (mmHg)	11 ± 2	11 ± 2	14 ± 4	14 ± 3	15 ± 3	14 ± 3	15 ± 4*	*0.28*
APP (mmHg)	72 ± 6	75 ± 9	58 ± 4	57 ± 11	68 ± 13	76 ± 6	66 ± 20	*0.89*
Cardiac rate (bpm)	83 ± 6	83 ± 15	101 ± 32	109 ± 23	108 ± 24	96 ± 18	93 ± 11*	*0.16*
Cardiac output (L/min)	2.7 ± 0.4	2.6 ± 0.5	2.5 ± 0.5	2.7 ± 1.4	2.4 ± 1.5	1.7 ± 0.3	2.1 ± 0.8	*0.41*
*Control group*								
MAP (mmHg)	78 ± 15	82 ± 9	69 ± 5	75 ± 3	81 ± 11	84 ± 11	79 ± 11	*0.76*
SAP (mmHg)	96 ± 13	101 ± 8	88 ± 6	93 ± 4	97 ± 8	101 ± 9	100 ± 13	*0.35*
DAP (mmHg)	64 ± 16	66 ± 9	54 ± 5	62 ± 3	68 ± 12	70 ± 12	65 ± 15	*0.97*
PAPmean (mmHg)	19 ± 1	31 ± 5	32 ± 6	36 ± 10	38 ± 11	36 ± 9	3 ± 9	*0.59*
PAOP (mmHg)	12 ± 2	13 ± 2	15 ± 2	16 ± 2	16 ± 2	16 ± 2	16 ± 2	*0.78*
CVP (mmHg)	8 ± 1	9 ± 2	10 ± 2	10 ± 2	11 ± 2	11 ± 1	11 ± 2	*0.08*
IAP (mmHg)	10 ± 3	11 ± 3	13 ± 4	13 ± 3	13 ± 5	12 ± 3	11 ± 3	*0.28*
APP (mmHg)	68 ± 15	71 ± 8	57 ± 6	62 ± 3	68 ± 10	72 ± 9	68 ± 12	*0.89*
Cardiac rate (bpm)	84 ± 12	92 ± 9	106 ± 16	124 ± 19	116 ± 18	116 ± 14	118 ± 14	*0.16*
Cardiac output (L/min)	2.4 ± 0.3	2.6 ± 0.4	2.3 ± 0.4	2.1 ± 0.4	1.9 ± 0.5	2.0 ± 0.5	2.0 ± 0 5	*0.41*

Hemodynamic parameters measured at the baseline and every hour during experiments in the two groups. Data are reported as mean; standard deviation. Statistically significant difference (*p* < 0.05) between the two groups found with multiple t-tests is reported as a * near to the Intervention Group values

ANOVA analysis *p values* are reported in the dedicated columns next to the Edema group measurements

*MAP* mean arterial pressure, *SAP* systolic arterial pressure, *DAP* diastolic arterial pressure, *PAPmean* pulmonary artery pressure (mean value), *PAOP* pulmonary artery occlusion pressure, *CVP* central venous pressure, *IAP* intra-abdominal pressure, *APP* abdominal perfusion pressure, *mmHg* millimeter mercury, *bpm* beat per minute, *L/min* liters per minute.

**Table 2:** Respiratory and blood gas analysis parameters

	Baseline	1st hour	2nd hour	3rd hour	4th hour	5th hour	6th hour	ANOVA (*p* value)
*Edema group*								
Airway pressure (peak)	12.0 ± 3.2	15.3 ± 3.4	20.8 ± 4.8	21.8 ± 4	21.8 ± 3.8*	22.2 ± 3.9	23 ± 4.9	*0.10*
Airway pressure (mean)	10.2 ± 4.9	13.7 ± 8.7	18.2 ± 8.6	18.5 ± 8.5	18.5 ± 8.5	18.7 ± 8.3	18.8 ± 8.1	*0.82*
EtCO_2_ (mmHg)	36.2 ± 2.7	37.9 ± 2.6	41.1 ± 3.4	42.3 ± 2.6	40.4 ± 3.4	39.4 ± 4.9	38.1 ± 5.9	*0.66*
pH	7.5 ± 0.1	7.4 ± 0.1	7.4 ± 0.1	7.4 ± 0.1	7.4 ± 0.1	7.4 ± 0.1	7.4 ± 0.1	*0.87*
pO_2_ (kPa)	35.1 ± 2*	31.6 ± 8.1	33.5 ± 5.5	34.6 ± 11.6	33.7 ± 9.5	29.1 ± 4.8	27.2 ± 8.9	*0.16*
pCO_2_ (kPa)	5.3 ± 0.4	5.7 ± 0.4	6.2 ± 0.5	6.2 ± 0.2	6.0 ± 0.2	6.0 ± 0.2	6.2 ± 0.4	*0.56*
Lactate (mmol/L)	1.8 ± 0.6	2.1 ± 0.5	2.5 ± 0.5	3.2 ± 1.3	3.4 ± 1.6	3.3 ± 1.6	2.6 ± 1.4	***0.04***
Hb (gr/dL)	79.0 ± 7.0	84.0 ± 7.0	84.0 ± 4.0	86.0 ± 6.0	89.0 ± 7.0	89.0 ± 9.0	85.0 ± 10.0	*0.12*
SpO_2_ (%) venous	59.3 ± 3.6	60.1 ± 7.7	55.2 ± 11.2	51.9 ± 14.7	48.5 ± 16.6	44.2 ± 15.1	44.5 ± 9.0	*0.21*
*Control group*								
Airway pressure (peak)	14.4 ± 3.0	17.0 ± 5.0	25.0 ± 9.0	27.0 ± 4.8	26.9 ± 4.1	26.9 ± 4.8	26.6 ± 4.6	*0.10*
Airway pressure (mean)	9.0 ± 3.6	9.9 ± 4.2	17.3 ± 5.6	18.4 ± 5.2	18.7 ± 5.3	18.7 ± 4.8	18.9 ± 5.1	*0.82*
EtCO_2_ (mmHg)	35.4 ± 2.7	37.2 ± 3.0	40.1 ± 4.5	41.5 ± 4.3	40.6 ± 4.3	43.9 ± 4.2	42 ± 4.1	*0.66*
pH	7.5 ± 0.1	7.4 ± 0.1	7.4 ± 0.1	7.4 ± 0.1	7.4 ± 0.1	7.3 ± 0.1	7.3 ± 0.1	*0.87*
pO_2_ (kPa)	24.4 ± 7.6	28.5 ± 5.4	32.1 ± 5.0	29.9 ± 8.6	29.5 ± 7.7	27.1 ± 7.0	28.4 ± 7.0	*0.16*
pCO_2_ (kPa)	5.1 ± 0.4	5.5 ± 0.7	5.8 ± 0.7	6.0 ± 0.5	5.9 ± 0.5	6.2 ± 0.7	6.4 ± 0.7	*0.56*
Lactate (mmol/L)	1.5 ± 0.4	1.9 ± 0.4	2.0 ± 0.4	2.2 ± 0.6	2.2 ± 0.6	2.1 ± 0.7	1.8 ± 0.5	***0.04***
Hb (g/dL)	81.0 ± 14.0	91.0 ± 12.0	93.0 ± 11.0	94.0 ± 8.0	94.0 ± 9.0	97.0 ± 11.0	94.0 ± 11.0	*0.12*
SpO_2_ (%) venous	50.7 ± 11.0	60.1 ± 5.1	51.7 ± 3.6	45.3 ± 6.2	38.3 ± 7.4	38.1 ± 6.0	39.3 ± 5.7	*0.21*

Respiratory and blood gas analysis parameters measured at the baseline and every hour during experiments in the two groups. Data are reported as mean ± standard deviation

Airways pressure (peak and mean) is reported in cmH_2_O

Statistically significant difference (*p* < 0.05) found with multiple comparison between the two groups are reported as a * near the Intervention Group values

ANOVA analysis *p values* are reported in the dedicated columns next to the Edema group measurements (*p* values < 0.05 are reported in bold)

*pO*_*2*_ partial pressure of oxygen in arterial blood, *pCO*_*2*_ partial pressure of carbon dioxide in arterial blood, *Hb* hemoglobin, *spO*_*2*_ oxygen saturated hemoglobin in ven ous blood (jugular vein).

Baseline measurements were similar in both groups and hemodynamic was similar between the two groups throughout the experiments. After starting endotoxin infusion, the systolic blood pressure decreased by 40% of the baseline value in all animals (minimum systolic pressure measured: 49 ± 4 in the Edema Group and 46 ± 9 in the Control Group). Once that value was reached, a noradrenaline infusion was started and titrated to sustain a mean arterial pressure (MAP) higher than 65 mmHg, as recommended by the Surviving Sepsis Campaign Guidelines [14]. The maximum infusion rate was 0.2 mcg/kg/min in the Edema group and 0.15 mcg/kg/min in the Control group, otherwise the total amount of noradrenaline infused throughout the study was similar in both groups.

The Edema group received 500 ml of Ringer acetate more than the Control group throughout the experiment. The fluid balance was + 740 ml in the Edema group and + 220 ml in the Control group (*p* < 0.05).

All the animals were ventilated using the same set up: *V*_T_ of 6 mL kg^−1^, *I*:*E* 1:2, FiO_2_ 0.5, RR 25 cycles/min and PEEP 5 cmH_2_O, which was gradually increased till 15 cmH_2_O during the first hour of observation.

Lactate value was found higher in the Edema group, compared to the Control group.

### Edema

Wet–dry weight was found higher in the Edema group in the lower lobes of lungs, in the kidneys (assessed together). In the duodenum Wet–dry weight showed higher number without a statistical difference (*p* = 0.06).

**Fig. 2:**
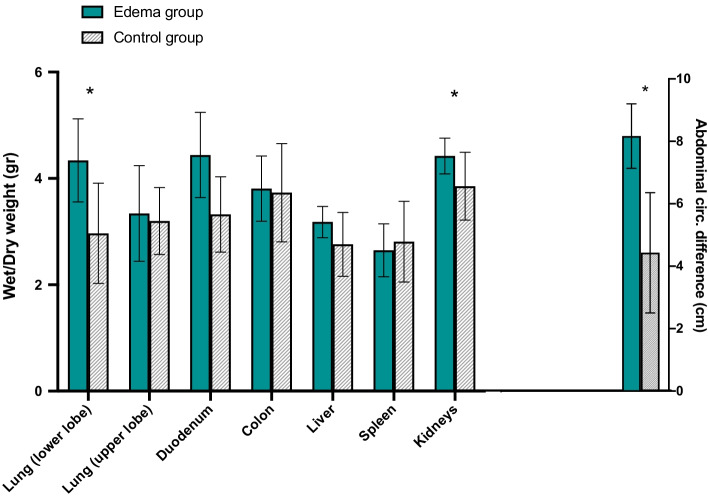
Wet–dry weight and abdominal circumference difference

Abdominal circumference (difference between the measurement at the end of the experiment and at baseline) was greater in the Edema group (+ 8.2 cm, on average, vs + 4.4 in the Control group) (see Fig. 2).

**Fig. 3:**
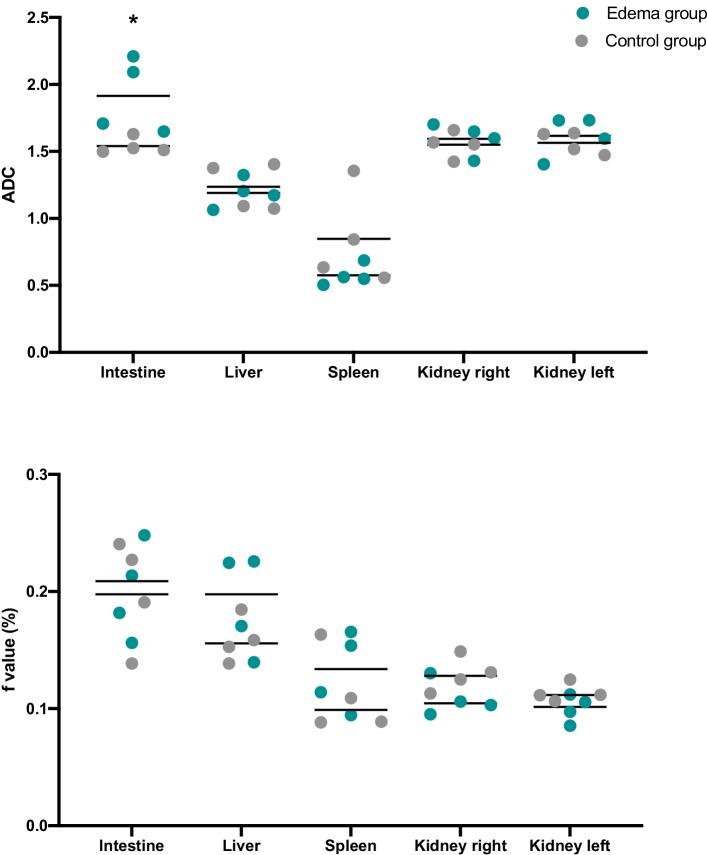
Data from DW-MR images acquisition. ADC is represented as an adimensional number, *f* value is represented as a percentage of the total amount of water in the tissue. *p < 0.05

ADC values were higher in the duodenum of the Edema group. The kidneys’ ADC value was similar in both groups and tended to correlate with the urinary output (*r* = 0.55). See Fig. 3.

**Fig. 4:**
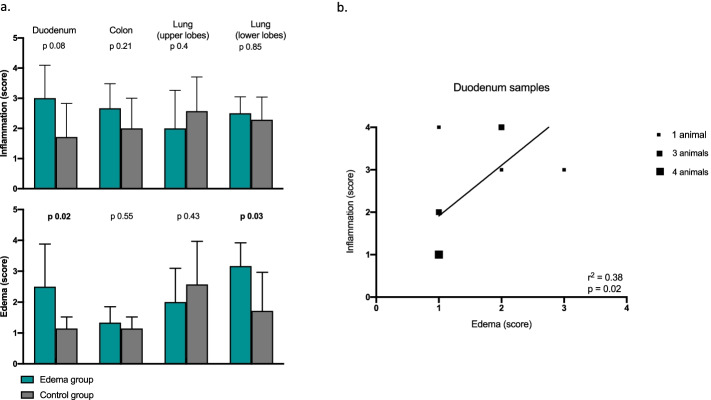
**a** Acute inflammation and edema scores in duodenum, colon and lungs samples (from upper and lower lobes) compared using Mann–Whitney test. *p *values are reported for every comparison. **b** Correlation (Spearman’s coefficient) between inflammation and edema score in duodenum. The square dimension increases with the number of animals that had the same scores

Histopathological edema score was higher in the Edema group than in the Control group in duodenum and lungs (lower lobes) samples. No difference in edema was found in samples from the colon and the upper lobes of the lungs (see Fig. 4).

### Perfusion

The *f *value was similar in both groups in the duodenum, the liver, and the spleen. In both kidneys, evaluated separately, the *f *value was numerically higher in the Control group than in the Edema group without a statistical difference (*p* = 0.09 in both kidneys). The same result was found when the two kidneys were analyzed together (see Fig. 3).

### Inflammation

**Fig. 5:**
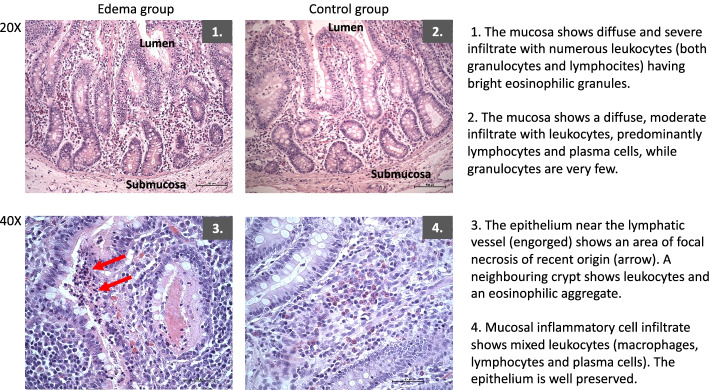
Histopathological samples from duodenum. Even if not all the animals showed the same degree of edema and inflammation as those in the figure, the presented slides are representative and illustrate patterns found throughout the groups. In the 20 × magnification the difference in the inflammation degree (the amount and type of infiltrate) is shown. In the 40 × magnification it is possible to see some recurrent pattern in the Edema group, such as engorged lymphatic vessel and areas of focal necrosis

Inflammation assessed by histopathology underlined a numerically higher inflammation in duodenum samples (*p* = 0.08) and the inflammation score correlated to the edema score (see Figs. 4 and 5). No differences were found in histopathological acute inflammation scores between the groups in other organs.

The cytokines concentration results are summarized in Table 3.

**Table 3:** Cytokines concentration, comparison between the two groups, at baseline and at the end of experiment (6th hour)

Cytokine concentration (pg/ml for ascites and blood samples; pg/mg of protein for tissues’ samples)														
		Arterial blood	Pulm. Art. blood	Portal vein blood	Hepatic blood	Lungs (upper lobe)	Lungs (lower lobe)	Duodenum	Colon	Liver	Spleen	Kidney right	Kidney left	Ascites
*IL6 concentration*														
Edema group	Baseline	1.65 ± 0.44	1.37 ± 0.85	1.87 ± 0.48	1.29 ± 0.87	//	//	//	//	//	//	//	//	//
	6th hour	3.99 ± 0.18	4.02 ± 0.15	4.10 ± 0.14	4.05 ± 0.15 *	4.08 ± 0.37	4.09 ± 0.36	**3.83 ± 0.26** **	3.92 ± 0.4	4.31 ± 0.24	3.92 ± 0.4	4.72 ± 0.16	4.67 ± 0.23	3.83 ± 0.6
Control group	Baseline	1.31 ± 0.96	1.24 ± 0.97	1.32 ± 0.85	1.73 ± 1.26	//	//	//	//	//	//	//	//	//
	6th hour	3.56 ± 0.59	3.63 ± 0.53	3.62 ± 0.64	3.38 ± 0.65	3.69 ± 0.36	3.46 ± 0.16	3.46 ± 0.16	3.47 ± 0.38	4.30 ± 0.10	3.73 ± 0.3	4.73 ± 0.15	4.96 ± 0.18	4.17 ± 0.06
*TNFα concentration*														
Edema group	Baseline	1.95 ± 0.15	1.94 ± 0.13	2.00 ± 0.11	2.00 ± 0.15	//	//	//	//	//	//	//	//	//
	6th hour	3.57 ± 0.16	3.58 ± 0.13	3.61 ± 0.15	**3.67 ± 0.18** *	3.57 ± 0.23	3.62 ± 0.24	3.78 ± 0.30	3.54 ± 0.21	4.19 ± 0.22	3.54 ± 0.21	4.41 ± 0.22	4.39 ± 0.21	4.09 ± 0.39
Control group	Baseline	1.99 ± 0.09	1.98 ± 0.10	2.10 ± 0.06	2.24 ± 0.53	//	//	//	//	//	//	//	//	//
	6th hour	3.20 ± 0.58	3.21 ± 0.58	3.15 ± 0.70	2.97 ± 0.74	3.57 ± 0.16	3.91 ± 0.37	3.63 ± 0.05	3.56 ± 0.29	4.34 ± 0.14	3.47 ± 0.22	4.59 ± 0.17	4.60 ± 0.22	4.08 ± 0.16
*IL1b concentration*														
Edema group	Baseline	1.02 ± 1.12	0.80 ± 0.10	0.89 ± 1.23	1.00 ± 1.20	//	//	//	//	//	//	//	//	//
	6th hour	2.55 ± 0.33	2.77 ± 0.23	2.75 ± 0.42	2.21 ± 1.12	3.46 ± 0.28	3.41 ± 0.32	3.65 ± 0.47 *	3.37 ± 0.52	4.44 ± 0.22	3.46 ± 0.52	4.69 ± 0.28	4.66 ± 0.27	1.00 ± 1.10
Control group	Baseline	1.34 ± 1.01	2.02 ± 1.06	1.39 ± 1.21	1.05 ± 1.05	//	//	//	//	//	//	//	//	//
	6th hour	2.47 ± 0.36	2.10 ± 0.97	1.95 ± 1.18	2.19 ± 1.05	3.39 ± 0.35	3.77 ± 0.28	3.20 ± 0.23	3.15 ± 0.31	4.28 ± 0.18	3.80 ± 0.43	4.57 ± 0.15	4.56 ± 0.09	0.93 ± 1.17

Measure unit: pg/ml for ascites and blood samples and pg/mg of protein for tissues’ samples

Values reported are Log10 of the concentrations (mean ± standard deviation). Statistical differences found with multiple comparison are reported as * (*p* < 0.05) and ** (*p* < 0.01)**,** both placed in the Edema group cell (the text is in bold to make the identification of differences between the groups easier)

As an additional analysis, cytokines concentration in organs’ tissues, blood and ascites was tested for correlation to IAP and other hemodynamics parameters (such as MAP and APP) measured at 6th hour of observation.

IL6 concentration correlated with IAP in the duodenum, the colon, the spleen and the left kidney (*p* < 0.05); IL6 was also correlated with IAP in the hepatic serum. *r*^2^ was 0.3 in liver, 0.26 right kidney.

TNFα concentration correlated with IAP in the lower lung lobes (*p* < 0.05); *r*^2^ was 0.28 in the colon and 0.24 in ascites.

TNFα correlated with MAP in the upper lung lobes and the colon. No other correlation between inflammation markers and hemodynamic parameters was identified.

## Discussion

The presented study showed that an impaired thoracic duct function can produce an increase in the duodenum’s edema which leads to an increase in acute inflammation and inflammatory marker production, even when perfusion is maintained in endotoxemic animals.

Together with the thoracic duct ligation [20, 21] endotoxin was infused in all the animals, as previous studies demonstrated that sepsis-like status induces an increase in the thoracic duct flow as a consequence of fluid extravasation [22, 23]. The ligation of the thoracic duct in otherwise healthy patients is not associated to a dramatic increase in abdominal edema in a short period [24], so the endotoxin infusion was a precondition for the ligation to produce a sensitive difference between the groups. Besides, sepsis is a frequent disorder in the intensive care, as the conditions that could reduce the abdominal lymphatic function; the induction of sepsis contributed to the plausibility of the model. The model used in the presented study is well-established and was used in previously to assess the edema [8, 9], inflammation [25, 26] and immunomodulation’s effect [27, 28] of different treatment and procedures.

The Edema group—with thoracic duct ligated—showed a higher edema in the duodenum, both calculated with MRI (ADC value [29]) and histopathological analysis (while wet–dry ratio gave a non-significant difference). Interestingly, colon showed no difference in the amount of edema. Lower lobes of lungs resulted more edematous as well, but upper lobes showed no differences. It is possible that the ligation of the abdominal portion of the thoracic duct also influenced the drainage from the lower part of the lungs, or that the alternative drainage system [30] was engorged.

Wet–dry ratio in the kidneys was higher in the Edema group as well, but ADC was similar in both groups. However, it is crucial to consider that in the kidneys this measurement correlates with the glomerular filtration rate, with the diuresis, and with the total amount of water content, as shown in the presented study in agreement with previous studies [31] (a weak correlation was found between diuresis and ADC values in both kidneys).

The increase in abdominal circumference at the end of the observation was used as a rough measurement of the ascites amount. The amount of ascites was not directly measured because of difficulties in finding an accurate technique, but the bigger increase in the abdominal circumference found in the Edema group reads for a higher amount of fluid.

Intra-abdominal pressure (IAP) increased more in the Edema group by the end of the experiment. The difference was small and with a limited clinical significance, but it could be related to the increase amount of ascites and edema. Considering the increase in the abdominal circumference the small increase in IAP reads for a high compliance of the abdominal compartment.

During the protocol, noradrenalin was used to maintain a MAP > 65 mmHg and to maintain a normal perfusion in the abdominal organs. As in sepsis-like state perfusion can be affected even when hemodynamics is sustained, and as edema could have had an effect on perfusion itself, MRI was primarily used to check regional perfusion of abdominal organs, which showed to be similar in both groups, except for kidneys that were less perfused in the Edema group compared to the controls. The increased ascites amount and IAP could have affected renal perfusion by the end of the observation period reducing glomerular filtration rate able to mask the edema component of ADC; that would explain the increased wet–dry weight with no increase in ADC.

The fact that both groups had similar small intestine/duodenum perfusion allowed the observation of the effect of edema per se on inflammation.

Acute inflammation assessed in the duodenum by histopathological analysis and IL6 and IL1b measurements was higher in the Edema group. Even if the histopathological inflammation score did not differ between the groups (*p* = 0.08), the pathologist found specific patterns throughout the edema group (see Fig. 5); besides, the inflammation score correlated with the edema score in duodenum samples, making clearer the association between edema and inflammation.

No other organs showed an increased inflammation in the Edema group, interestingly lungs’ lower lobes showed similar level of inflammation in spite of a higher edema level.

The duodenum showed a higher sensitivity to an acute impairment of the thoracic duct function and a higher inflammatory response to the increase of edema, compared with any other abdominal organs and with lungs.

Even if the difference in IAP was found only at the end of the study and it was a small one, IL6 correlated with IAP in most of the blood samples and organs. In previous studies [10, 32] IAP was found correlated with TNFα better than with IL6 when a higher edema formation was associated to a worse perfusion and hemodynamics and not with a decrease in edema clearance. Thus, it is possible that TNFα induces edema and increased ascites production, leading to increased IAP, but that edema per se does not explicitly trigger TNFα production [1].

Further studies are needed to investigate the effect of an acute impairment of the abdominal lymphatic drainage in humans, but the results of the presented study suggest that in clinical situations with a potential thoracic duct’s function deterioration (as high thoracic pressures applied by mechanical ventilation, or extensive thoracic trauma) a specific attention should be paid to the small intestine and its possible inflammation-triggering role.

### Limitations of the study

The presented study is affected by a number of limitations. First, this is an animal study with all the inherent limitations, yet still relevant as a model of human disease. Second, the small number of animals included in the study could have prevented the identification of differences between the two groups. Moreover, the number of animals that were MRI examined was even smaller. The thoracic duct ligation technique has been used in the literature, but it is still a fairly new technique for the induction of abdominal edema.

The observation period was 6 h long; a longer observation time could have shown a greater increase in abdominal edema in other organs than duodenum. However, our primary aim was to investigate the short-time effect of a decreased lymphatic clearance caused by an acute impairment of the thoracic duct function in a systemic inflammatory state.

## Conclusions

Our experimental study showed that ligating the thoracic duct can induce an increase of edema in the duodenum and an increase in ascites. The increased edema of the duodenum was associated to an increase in inflamattion, even when perfusion was maintained.

## Data Availability

The datasets used and/or analyzed during the current study are available from the corresponding author on reasonable request.

## Abbreviations

ADC: Apparent diffusion coefficient
APP: Abdominal perfusion pressure
DW-MRI: Diffusion-weighted magnetic resonance imaging
ELISA: Enzyme-linked immuno sorbent assay
IAP: Intra-abdominal pressure
IL1b: Interleukin 1 b
IL6: Interleukin 6
MAP: Mean arterial pressure
MRI: Magnetic resonance imaging
PEEP: Positive end-expiratory pressure
TNFα: Tumor necrosis factor α
*V*_T_: Tidal volume
